# Instrument referral criteria for PlusoptiX and SureSight based on 2021 AAPOS guidelines: A population-based study

**DOI:** 10.3389/fpubh.2022.959757

**Published:** 2022-09-26

**Authors:** Qi Yan, Rui Li, YingXiao Qian, Xiao Lin, Hui Zhu, Yue Wang, Xiaoyan Zhao, Xiaohan Zhang, Qigang Sun, Qingfeng Hao, Haohai Tong, Yue Zhu, Zhitong Li, Yan Zhu, Hu Liu, Dan Huang

**Affiliations:** ^1^Department of Ophthalmology, The First Affiliated Hospital With Nanjing Medical University, Nanjing, China; ^2^College of Optometry, University of Houston, Houston, TX, United States; ^3^Department of Ophthalmology, The Affiliated Changzhou No. 2 People's Hospital of Nanjing Medical University, Changzhou, China; ^4^Department of Ophthalmology, Wuxi Children's Hospital, Wuxi, China; ^5^Department of Ophthalmology, Nanjing International Hospital, Nanjing, China; ^6^The Second Affiliated Hospital, Zhejiang University School of Medicine, Eye Center, Hangzhou, China; ^7^Fourth School of Clinical Medicine of Nanjing Medical University, Nanjing, China

**Keywords:** amblyopia risk factors, vision screening, failure criteria, referral criteria, AAPOS 2021

## Abstract

**Objective:**

The study aims to assess two refractive instrument-based methods of vision screening (SureSight and PlusoptiX) to detect refractive amblyopia risk factors (ARFs) and significant refractive errors in Chinese preschool children and to develop referral criteria according to the 2021 AAPOS guidelines.

**Methods:**

Eye examinations were conducted in children aged 61 to 72 months (*n* = 1,173) using a PlusoptiX photoscreener, SureSight autorefractor, and cycloplegic retinoscopy (CR). The Vision Screening Committee of AAPOS's preschool vision screening guidelines from 2021 were adopted for comparison. Paired *t*-test analysis and Bland–Altman plots were used to assess the differences and agreement between the PlusoptiX photoscreener, SureSight autorefractor, and CR. In addition, the validity of the cut-off values of the several ARFs measured with the SureSight and PlusoptiX was estimated using receiver operating characteristic (ROC) curves and compared to the age-based 2021 AAPOS examination failure levels.

**Results:**

A total of 1,173 children were tested with comprehensive eye examinations. When the referral numbers based on the 2013 (43/3.67%) and 2021 (42/3.58%) AAPOS guidelines were compared, significant differences between the values of astigmatism (72.09 vs. 52.38%) and anisometropia (11.63 vs. 38.10%) were found. The 95% limits of agreement (LOA) of the spherical value and the cylindrical value between PlusoptiX and CR were 95.08 and 96.29%. It was 93.87 and 98.10% between SureSight and CR. Considering refractive failure levels, the ROC curves obtained the optimal cut-off points. However, the PlusoptiX and the SureSight showed lower efficiency in hyperopia (Youden index, 0.60 vs. 0.83) and myopia (Youden index, 078 vs. 0.93), respectively. After adjusting the above cut-off points, the optimized NES (Nanjing Eye Study) referral criteria for myopia, hyperopia, astigmatism, and anisometropia were –0.75, 1.25, –1.0, and 0.5 with PlusoptiX and –1.25, 2.75, –1.5, and 0.75 with SureSight.

**Conclusions:**

SureSight and PlusoptiX showed a good correlation with CR and could effectively detect refractive ARFs and visually significant refractive errors. There were obvious advantages in detecting hyperopia using SureSight and myopia using PlusoptiX. We proposed instrumental referral criteria for age-based preschool children based on AAPOS 2021 guidelines.

## Introduction

Amblyopia is a common neurodevelopmental vision disorder with at least 1–2% prevalence (1, 2). Amblyopia not only compromises visual acuity but also contrast sensitivity, stereopsis, and motion perception. Early interventions for amblyopia show promisingly high cure rates. However, the efficacy decreases with age (3). Amblyopia risk factors (ARFs) include amblyogenic factors, such as strabismus, anisometropia, refractive error, and vision deprivation, and they can interrelate with each other (3). Therefore, the US Preventive Services Task Force (USPSTF) recommends that children aged 3 to 5 years could be screened for ARFs at least once (4). The American Association for Pediatric Ophthalmology and Strabismus (AAPOS) published the AAPOS 2021 guidelines, dividing the targets for vision screening into ARFs and visually significant refractive errors by age. In addition, the AAPOS 2021 guidelines adjust refractive thresholds to reduce over-referral and provide new guidance and requirements for revising instrument referral criteria.

Current screening methods based on a visual acuity chart may have limited accuracy due to children's poor cooperation and examiners' lack of experience (3, 5). Instrument-based screening uses autorefraction or photorefraction, which identifies the presence and magnitude of refractive error instead of measuring visual acuity. Therefore, compared to a visual acuity chart, instrument-based vision screening is quicker to administer as it requires minimal child cooperation. The American Academy of Pediatrics recommends instrument-based vision screening when available (6). Therefore, some user-friendly vision screeners have been employed.

The SureSight Vision Screener has been used as a handheld autorefractor for years, especially in developing countries, with well-established refractive error referral criteria. The PlusoptiX Photoscreener is also used as one of the photorefraction devices. Both devices are now being used simultaneously in different medical institutions in many regions. However, a few studies have compared the two devices and validated their accuracy. Meanwhile, the optimum refractive error referral criteria for two devices are yet to be determined based on 2021 AAPOS guidelines.

Based on the updated preschool vision screening guidelines from the Vision Screening Committee of AAPOS for 2021, we evaluated the accuracy of the SureSight Vision Screener and the PlusoptiX Photoscreener in detecting refractive ARFs and visually significant refractive errors. In addition, we tried to develop and optimize customized instrument referral criteria based on the Nanjing Eye Study (NES).

## Materials and methods

### Study population

NES is a population-based cohort study aiming to longitudinally observe the onset and progression of childhood ocular diseases in eastern China. As previously described (7), the children born between September 2011 and August 2012 in Yuhuatai District, Nanjing, China, who entered a kindergarten in Yuhuatai District were invited to participate in NES to undergo comprehensive eye examinations annually. The primary data presented in this paper were obtained in 2017 when these children were 60 to 72 months old.

### Examinations

A team of six trained ophthalmologists and four optometrists conducted a comprehensive eye examination on all participants. Children's roster and basic information, including name, sex, and birth date, were obtained from each kindergarten's principal and were checked during the examination. Examinations including anthropometric parameters, distance visual acuity (VA, including uncorrected visual acuity, UCVA; presenting visual acuity, PVA and best corrected visual acuity, BCVA), anterior segment and fundus examination, instrument-based vision screening using automated technology, table-mounted autorefractor before and after cycloplegia, stereo acuity test, ocular alignment and motility, ocular biometric parameters, intraocular pressure, accommodative response, and optical coherence tomography were performed in the setting of every kindergarten. In addition, instrument-based vision screening was performed using automated technology before cycloplegia, including a handheld autorefractor (SureSight, Welch-Allyn, Inc, Skaneateles Falls, NY) and handheld photoscreener (PlusoptiX A12C, PlusoptiX GmbH, Nuremberg, Germany). Children with suspected or confirmed eye problems were referred to senior ophthalmologists and underwent further examinations.

The SureSight Vision Screener was placed 35 cm in front of the children. When the child is attracted by a circle of eight flashing green LEDs surrounding a small, central red light, the device measures refractive error monocularly along two meridians. After both eyes were measured individually, the SureSight displayed refractive values of both eyes and confidence numbers. The confidence number indicates the number of good readings obtained and their consistency, ranging from 1 to 9. The manufacturer's recommended minimum confidence number is 6. As recommended by the manufacturer for children younger than age 6 years, the SureSight was used in the “Child” mode, which adds a constant to the sphere value obtained, to correct the accommodative response of the non-cycloplegic child during testing. The spherical value ranged from –5.00D to +7.00D, and the cylindrical value ranged from –3.00D to + 3.00D. A +9.99 or −9.99 indicates a reading outside the unit's measurement range.

The PlusoptiX photoscreener was placed in front of the children at a distance of 1 meter under dim light, using a smiling face as a fixation target. The examination was performed simultaneously on both eyes while the non-cycloplegic child stared at the fixation target. This device's spherical and cylindrical value ranged from –7.00D to +5.00D in 0.25D increments, with asymmetry ranging from 0 to 25° in increments of 0.1°. The screener would show “Myopia” or “Hyperopia” directly when the spherical equivalent (SE) was over range. The test was performed at least 10 times until success.

Cycloplegic refraction was performed on (1) voluntary children and (2) children with suspected or confirmed eye problems, using retinoscopy. One drop of topical 1.0% cyclopentolate (Cyclogyl, Alcon Pharmaceuticals) was administered to each eye two times at a 5-min interval. After 15 min, the third drop of cyclopentolate was administered if the pupil size was <6 mm or the pupillary light reflex was still present.

### Definition

Defined by the updated 2021 AAPOS guidelines, cycloplegic confirmatory examination failure levels for children aged >48 months should detect myopia >-2.00 D, hyperopia >4.00 D, astigmatism >-1.75 D, and anisometropia >1.25 D (8).

### Data analysis

Except for the anisometropia calculation, only data for the right eye was analyzed to avoid enantiomorphism bias. Therefore, SE was calculated as spherical plus half of the cylindrical error. Consistent with AAPOS 2021 guidelines, we used the myopic meridional refractive power for myopic refractions, the hyperopia meridional refractive power for hyperopic refractions, and the magnitude of the difference of the lesser meridian for anisometropic determination (8).

Data analysis was performed using the IBM Statistical Package for the Social Sciences program V13.0 (SPSS, Chicago, IL, USA), and *P* < 0.05 was considered statistically significant. Descriptive data were presented as mean ± standard deviation (SD). Paired *t*-test analysis and Bland–Altman plots were used to assess the differences and agreement between the PlusoptiX photoscreener, SureSight autorefractor, and CR separately. The validity of the cut-off values of the several ARFs measured with the SureSight and with PlusoptiX was estimated by receiver operating characteristic (ROC) curves using CR as a reference and compared with the age-based 2021 AAPOS examination failure levels (8). The final optimized referral criteria, NES referral criteria, were obtained after adjusting the failure levels based on the ROC curve's different effectiveness of cut-off values. We calculated the sensitivity (Se), specificity (Sp), Youden index, positive predictive value (PPV), and negative predictive value (NPV) of NES referral criteria, Arnold referral criteria, and AAPOS 2021 referral criteria (using the numeric values of AAPOS 2021 failure criteria), which were compared to criterion standard confirmatory examinations by ophthalmologists (9).

## Results

A total of 1,609 children (aged 66.84 ± 3.39 months) were tested with instrument-based vision screening, including 889 boys and 720 girls. Cycloplegic refraction was performed in 1,173 children (72.90%, 1,173/1,609). According to referral criteria of 2013 AAPOS and 2021 AAPOS, 43/42 (3.67%/3.58%) children were confirmed to have refractive ARFs, including 4/6 (9.30%/14.29%) with myopia, 12/11 (27.91%/26.19%) with hyperopia, 31/22 (72.09%/52.38%) with astigmatism, and 5/16 (11.63%/38.10%) with anisometropia (Table 1). Several children had two or more different refractive ARFs and visually significant refractive errors. Both guidelines obtained similar referral rates (3.67%/3.58%), but the number of referrals decreased for astigmatism and increased for anisometropia based on AAPOS 2021. Nineteen children were defined as inconclusive screenings from whom SureSight (*N* = 10) and PlusoptiX (*N* = 10) could not get conclusive results. One child was inconclusive in both devices. Seven inconclusive screenings were true positive for SureSight, and only one child was true positive for PlusoptiX. Based on the available results, inconclusive screenings were not predictably associated with a high refractive error or ophthalmic diseases.

**Table 1:** Comparison of referrals based on the AAPOS 2013 and AAPOS 2021.

**ARFs and visually significant refractive errors**	**Cases present**	
	**2013**	**2021**
Hyperopia	12	11
Myopia	4	6
Astigmatism	31	22
Anisometropia	5	16
All refractive ARFs	43	42

**Figure 1:**
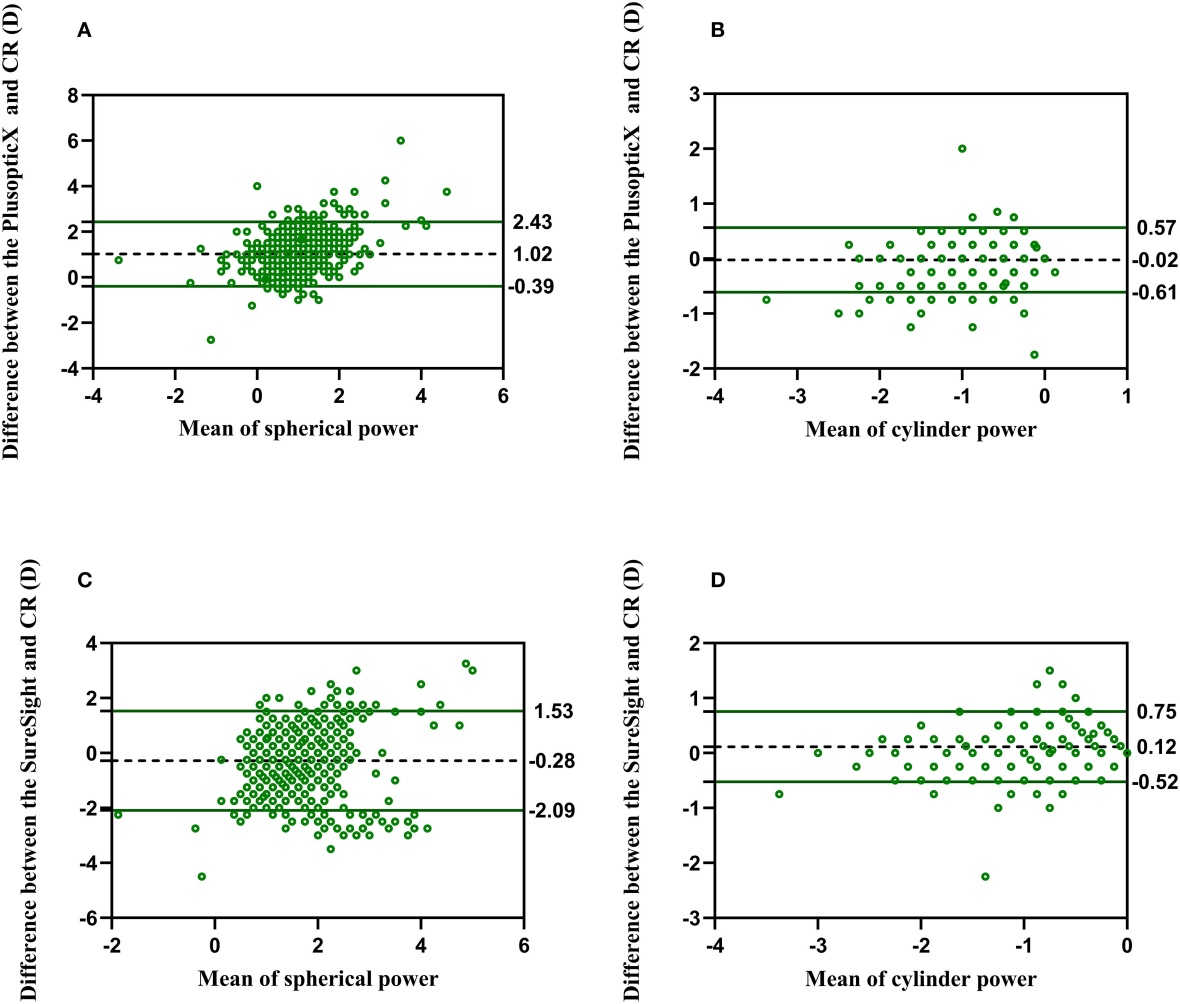
Bland–Altman plots show the agreement between PlusoptiX, SureSight, and cycloplegia retinoscopy. The difference between sphere and cylinder between **(A,B)** PlusoptiX and CR and **(C,D)** SureSight and CR.

Data of the right eye of 1,159 children were compared by paired *t*-test (Table 2) and Bland–Altman plots (Figure 1), consisting of 1,154 children with conclusive screenings and 5 children with inconclusive but numerical screenings of both devices. Compared with CR, PlusoptiX underestimated the mean cylindrical value (-0.42 ± 0.36 vs. –0.44 ± 0.45, average difference: –0.02 ± 0.30, *P* = 0.01) and the mean spherical value (0.34 ± 0.51 vs. 1.36 ± 0.78, average difference: 1.02 ± 0.72, *P* < 0.001). Statistical differences between SureSight and CR were found in mean cylindrical value (–0.55 ± 0.38 vs. –0.44 ± 0.45; average difference: 0.12 ± 0.33 D; *P* < 0.001) and the mean spherical value (1.64 ± 0.71 D vs. 1.36 ± 0.78 D; average difference: –0.28 ± 0.92 D; *P* < 0.001). According to the 95% LOA of the mean spherical value and the cylindrical value, the proportion of people within this range to the total was 95.08 and 96.29% between PlusoptiX and CR (Figures 1A,B), and was 93.87 and 98.10% between SureSight and CR (Figures 1C,D), respectively.

**Table 2:** Comparison of SureSight, PlusoptiX, and cycloplegic retinoscopy in 1,159 children.

	**Sphere (D)**					**Cylinder (D)**				
	**Mean**	**SD**	**95% CI**		***P***	**Mean**	**SD**	**95% CI**		***P***
CR	1.36	0.78	1.31	1.4	N/A	–0.44	0.45	–0.47	–0.41	N/A
The plusoptiX	0.34	0.51	0.31	0.37	N/A	–0.42	0.36	–0.44	–0.4	NA
SureSight	1.64	0.71	1.6	1.68	N/A	–0.56	0.39	–0.58	–0.53	N/A
CR-Plus	1.02	0.72	0.98	1.06	< 0.001	–0.02	0.3	–0.04	–0.01	0.01
CR-SureSight	–0.28	0.92	–0.34	–0.23	< 0.001	0.12	0.33	0.1	0.13	< 0.001

CR, Cycloplegic retinoscopy; D, Diopter; SD, Standard Deviation; CI, Confidence Interval; N/A, not applicable.

**Figure 2:**
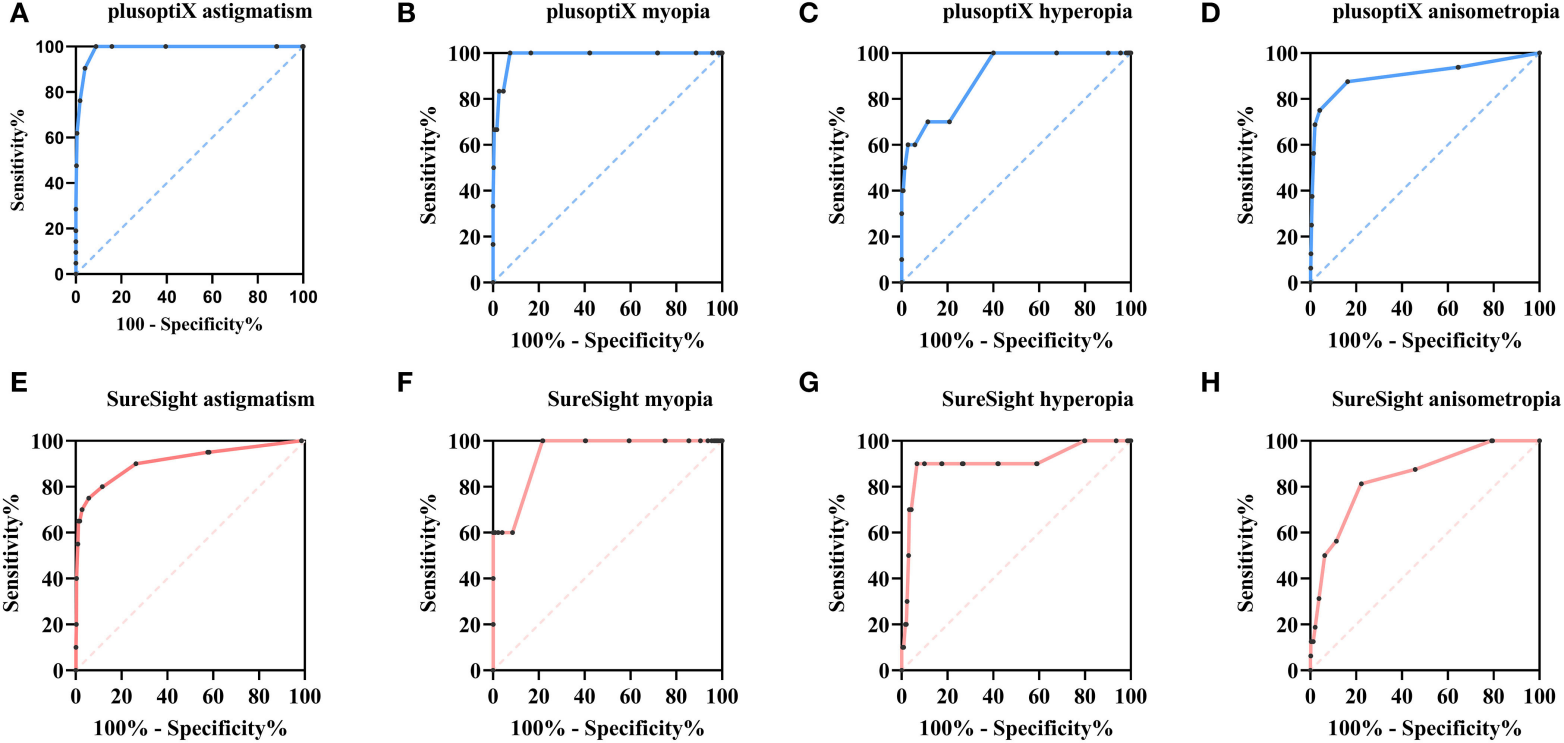
Receiver operating characteristic (ROC) curve for refractive results obtained by PlusoptiX and SureSight. ROC curve for **(A-D)** refractive results obtained by PlusoptiX and **(E-H)** refractive results obtained by SureSight.

We conducted ROC analyses according to the results with specific values for each screening tool tested against the presence/absence of refractive examination failure levels, and ROC curves are shown in Figures 2A–H. Table 3 provides the area under the curve (AUC), a measurement for comparing the screening tools' diagnostic benefits, sensitivity, specificity values, Youden index, and PPV. The PlusoptiX obtained a better AUC value and indicated better diagnostic power than the SureSight. The two instruments have higher accuracy in astigmatism (Youden index, plusoptiX = 0.91, SureSight = 0.92), which were the most common disorder (*N* = 22). Anisometropia (*N* = 16) gained a relatively poor Youden index (plusoptiX = 0.71 vs. SureSight = 0.59), on the contrary, myopia scored well (0.93 vs. 0.78), although it had the fewest positive screenings (*N* = 6). PlusoptiX had 100% sensitivity and lower specificity in detecting refractive factors except for anisometropia, and the screening effectiveness was inferior to the SureSight (Youden index: 0.60 vs. 0.83) in the aspect of hyperopia.

**Table 3:** Characteristics of ROC curves for refractive ARFs obtained by two refractive instrument-based methods compared with cycloplegic retinoscopy.

**ARFs**		**AUC**	**P**	**95% CI**	**Cut-off (D)**	**Se (%)**	**Sp (%)**	**Youden index**	**PPV (%)**	**NPV (%)**
Hyperopia (*N* = 11)	PlusoptiX	0.90	< 0.001	0.81	0.98	0.38	100.00	59.93	0.60	2.12	100.00
	SureSight	0.90	< 0.001	0.78	1.00	2.63	90.00	93.28	0.83	10.34	99.91
Astigmatism (*N* = 22)	PlusoptiX	0.99	< 0.001	0.98	1.00	–0.88	100.00	91.16	0.91	17.21	100.00
	SureSight	0.96	< 0.001	0.88	1.00	–1.38	95.24	97.13	0.92	37.74	99.91
Myopia (*N* = 6)	PlusoptiX	0.99	< 0.001	0.97	1.00	–0.63	100.00	92.57	0.93	6.52	100.00
	SureSight	0.94	< 0.001	0.87	1.00	0.56	100.00	78.39	0.78	1.95	100.00
Anisometropia (*N* = 16)	PlusoptiX	0.90	< 0.001	0.80	1.00	0.38	87.50	83.70	0.71	6.97	99.79
	SureSight	0.84	< 0.001	0.74	0.94	0.69	81.25	77.91	0.59	4.87	99.67

ROC, receiver operator characteristic curve; ARFs, amblyopia risk factors; AUC, area under the curve; Se, Sensitivity; Sp, Specificity; CI, Confidence Interval; D, Diopter; PPV, positive predictive value; NPV, negative predictive value.

Considering refractive failure levels based on ROC curves, the optimal cut-off points which corresponded to the maximum Youden index for myopia, hyperopia, astigmatism, and anisometropia with PlusoptiX were –0.63, 0.38, –0.88, and 0.38, and with SureSight were 0.56, 2.63, –1.38, and 0.69. Considering clinical practice and the low Sp (59.93%) and Youden index (0.60) of the best cut-off value for detecting hyperopia with PlusoptiX, we chose the second-best cut-off value of 0.88 (Sp = 88.55%, Youden index = 0.59) to replace 0.38. Then, we obtained a more appropriate instrument referral criteria (myopia ≤ –0.75, hyperopia ≥ 1.00, astigmatism ≤ –1.00, anisometropia ≥ 0.5), called NES referral criteria (Table 4). Similarly, we adjusted the cut-off value of myopia for the SureSight and obtained the corresponding NES referral criteria (myopia ≤ –1.25, hyperopia ≥ 2.75, astigmatism ≤ –1.50, anisometropia ≥ 0.75).

**Table 4:** Assessment and optimization of two refractive instrument-based methods for detecting refractive ARFs.

**Device**	**Referral criteria**	**Hyper**	**Myopia**	**Astig**	**Aniso**	**Se (%)**	**Sp (%)**	**Youden index**	**PPV (%)**	**NPV (%)**	**FPR(%)**	**FNR(%)**
PlusoptiX	AAPOS 2021	>4.00	< -2.00	< -1.75	>1.25	46.34	98.93	0.45	61.29	98.06	1.07	53.66
	Arnold referral criteria	≥3.00	≤ -2.50	≤ -2.50	≥1.75	24.39	99.38	0.24	58.82	97.29	0.62	75.61
	NES referral criteria	≥1.00	≤ -0.75	≤ -1.00	≥0.50	92.68	71.66	0.64	10.67	99.63	28.34	7.32
SureSight	AAPOS 2021	>4.00	< -2.00	< -1.75	>1.25	68.57	94.24	0.63	26.97	98.98	5.76	31.43
	NES referral criteria	≥2.75	≤ -1.25	≤ -1.50	≥0.75	88.57	71.99	0.61	8.93	99.51	28.01	11.43

Se, Sensitivity; Sp, Specificity; PPV, positive predictive value; NPV, negative predictive value; FPR, false positive rate; FNR, false negative rate.

Table 4 shows Se, Sp, PPV, and NPV for detecting refractive ARFs for the referral criteria of AAPOS 2021, Arnold referral criteria, and the NES referral criteria. Based on the referral criteria of AAPOS 2021, the Sp obtained were relatively high (PlusoptiX = 98.93%, SureSight = 94.24%). The AAPOS 2021 missed half of the diagnosis (Se = 46.34%) and showed poor screening efficiency (Youden index = 0.45) for the PlusoptiX, while SureSight performed better (Se = 68.57%, Youden index = 0.63). Se (24.39%) and the Youden index (0.24) of Arnold referral criteria declined significantly. Overall, NES referral criteria reduced missed diagnosis (Se = 92.68% for PlusoptiX, 88.57% for SureSight) and misdiagnosis (Sp = 71.66, 71.99%) and achieve acceptable screening efficiency (Youden index = 0.64, 0.61).

## Discussion

The present study assessed two refractive instrument-based methods of vision screening (SureSight and PlusoptiX) based on the 2021 AAPOS guidelines and proposed instrumental referral criteria for PlusoptiX (myopia ≤ –0.75, hyperopia ≥ 1.25, astigmatism ≤ –1.0, anisometropia ≥ 0.5) and SureSight (myopia ≤ –1.25, hyperopia ≥ 2.75, astigmatism ≤ –1.5, anisometropia ≥ 0.75), named NES referral criteria. The criteria were proposed recommendations for PlusoptiX and Suresight referral (manufacturer or user) based on AAPOS 2021 guidelines for the ARFs and visually significant refractive errors.

Instrument-based vision screening is appropriate for developing and populous countries where grassroots community medical personnel is short. Applying instrument- and age-specific pass/fail refractive error criteria based on the patient population, economic status and frequency of screening could improve the efficiency of screening. Two studies have compared Se and Sp of the PlusoptiX and the SureSight with CR and found that they were both reliable (10, 11). However, few studies have been conducted assessing their accuracy in the same population and improving referral criteria, especially based on the failure levels of updated AAPOS 2021, which significantly revised the threshold (8).

The 2013 and 2021 AAPOS guidelines got similar referrals (43 vs. 42) in the same population. The referrals showed a reduction in astigmatism (22/42 vs. 31/43) and an increase in anisometropia (16/42 vs. 5/43) based on the 2021 AAPOS guideline, which showed the effectiveness of threshold adjustment (8). The two instruments screened 10 children each who were classified as inconclusive screenings, and 70% for SureSight and 10% for PlusoptiX met failure levels, so we recommended referring inconclusive screenings, especially for SureSight (12, 13). Inconclusive screenings were not predictably associated with a high refractive error or ophthalmic diseases but may be attributable to covered pupils, poor cooperation, or inattention (13).

In the present study, most children were hyperopic. Compared with CR (1.36 ± 0.78D), PlusoptiX (0.34 ± 0.51D) underestimated the mean spherical value, and accommodation caused by fixation (PlusoptiX was performed simultaneously on both eyes) may result in this myopic tendency (14). In addition, the cylinder value was lower in PlusoptiX (–0.02 ± 0.30) and higher in SureSight (0.12 ± 0.33) than in CR. Our results suggested that SureSight overestimated astigmatism, consistent with other studies (12, 15).

Except for the sphere of SureSight, both instruments showed a good correlation with CR, and 95% LOA was more than 95%. One study showed that 95% LOA of SE between SureSight and CR was about 90% (16). As the previous study suggested, adding a constant factor to correct the fixation myopia induced by the SureSight may not solve the problem because each child's degree of accommodation is unforeseeable (17). We thought that this arbitrary addition of 1.50D may be one of the reasons why worse agreement and biases appear in the spherical value of SureSight.

In this population-based study, only 42 successfully tested children were confirmed to have refractive ARFs or visually significant refractive errors, thus increasing the difficulty in analyzing the screening accuracy. The AUC value of PlusoptiX was superior to SureSight for each refractive error, indicating that the former was more powerful and reliable in predicting refractive ARFs. We consider it difficult to precisely obtain myopia cut-off because the children tend to be hyperopia at an early age (14), especially for SureSight, which obtained a higher sphere value than CR. Only six children had myopia according to AAPOS 2021 guidelines, and the small number of positive cases could reduce screening efficiency (14). PlusoptiX, surprisingly, obtained a relatively meaningful myopia cut-off value. Some articles argued that PlusoptiX underestimated hyperopia and overestimated myopia in cases of normal accommodation (18, 19), which might result in a poor Youden index for hyperopia and a better Youden index for myopia. Contrary to expectations, the efficiencies of obtained anisometropia cut-off value were not good, which may be related to different calculation methods of anisometropia and the fewer true positive cases.

When proposing a new referral criterion, the best cut-off value (0.38D) of hyperopia for PlusoptiX resulted in numerous misdiagnoses (Sp = 59.93%). Considering that the PlusoptiX tended to underestimate hyperopia (18, 19), the hyperopia criterion was raised to 0.88, corresponding to the second largest of the Youden index. The SureSight worked well in astigmatism and hyperopia. Therefore, we adjusted the criteria of myopia, choosing the cut-off value with the highest Youden index in myopia refraction. As the severity of the anisometropia ARFs causes amblyopia (8, 20), we preferred not to modify the criterion. After replacing them with equivalent and more general values, we obtained the final NES referral criteria (Table 4).

The 2021 AAPOS guidelines are intended to be used with the gold-standard examination to identify true positives and to serve as a standard for comparison of referral criteria. For Suresight, the NES referral criteria got a similar Youden index to the referral criteria of AAPOS 2021 (0.61 vs. 0.63), as the former was more sensitive (88.57 vs. 68.57%) and got less specificity (71.99 vs. 94.24%). For PlusoptiX, the referral criteria of AAPOS 2021 trended to significantly miss ARFs and visually significant refractive errors (Se: 46.34 vs. 92.68%), supporting the recommendation that the 2021 AAPOS referral criteria not be used directly in photoscreening device criteria. NES referral criteria were more cautious and sensitive, screening for more positive cases but reducing the percentage of true positives. Some studies suggested that most children with ARFs do not develop amblyopia (21). Preschool vision screening for amblyopia requires attention to lower misdiagnosis and referral rates, especially in economically developed areas. However, if we fail to detect ARFs at the age of 5–6 years may delay the control and treatment of amblyopia (3, 4). Moreover, due to the high morbidity of myopia in China (22), the NES criteria met the basic requirements that the screening of children (>4 years) should focus on the control of myopia caused by near-distance learning. More sensitive and careful screening with NES referral criteria is more beneficial for these age-specific children.

However, it is unsuitable for comparing our results with relevant studies adopting the guidelines of AAPOS 2013. Therefore, a further study employing the 2021 referral guideline of AAPOS is warranted. Recently, Arnold et al. revised the instrument referral criteria of three photoscreeners based on the 2021 AAPOS guidelines, including PlusoptiX (9). In the present study, the NES referral criteria obtained the maximum efficiency and performed better than the Arnold referral criteria in Chinese eastern preschool children. The diverse consequences of the Arnold referral criteria may result from participant heterogeneity, including location, sample source and age, and device models. Altogether, the application of instrument- and age-specific referral criteria should consider the characteristics of the population, economic status, and frequency of screening.

The strength of this study included its large sample size of preschool children in a population-based study, a particular age range in a specific race, a large sample size of a cycloplegic refraction, the application of the updated AAPOS guidelines, relatively comprehensive indexes, and power of the test for refractive ARFs and visually significant refractive error with two refractive instrument-based methods.

The limitations of this study include the fact that 27% of children without cycloplegia may cause bias. Besides, SureSight has been discontinued for sale, although it is still used in many places. Furthermore, because of the small number of patients, particularly those with myopia, it was difficult to assess the cut-off value in this preschool population accurately. More research is needed to assess the cost-effectiveness, convenience, and accuracy of refractive instrument-based methods in children of various ages and races.

Nonetheless, this investigation revealed several valuable findings. First, the sphere and cylinder were consistent between the PlusoptiX and CR in preschool children aged 61–72 months. Second, there was no significant difference in the number of cases between the AAPOS guidelines from 2021 to 2013, but the latter focused more on anisometropia and reduced referrals for astigmatism. Third, there were obvious advantages in detecting hyperopia using SureSight and myopia using PlusoptiX. Finally, based on the AAPOS 2021 uniform guidelines, evidence-based instrument referral criteria derived from ROC curves were provided to children ≥4 years of age and would guide the screening of ARFs and visually significant refractive errors.

## Data availability statement

The raw data supporting the conclusions of this article will be made available by the authors, without undue reservation.

## Ethics statement

The studies involving human participants were reviewed and approved by the Ethics Committee of The First Affiliated Hospital with Nanjing Medical University. Written informed consent to participate in this study was provided by the participants' legal guardian/next of kin.

## Author contributions

HL and DH designed the study. QY, RL, and YXQ participated in manuscript preparation. QY and XL prepared tables and figures. QY, RL, and DH performed data interpretation and analysis. RL, YW, XZhao, XZhang, QS, QH, HT, YuZ, ZL, and YaZ performed the ocular examinations and questionnaire. All authors contributed to the article and approved the submitted version.

## Funding

This work is supported by the National Natural Science Foundation of China (Nos. 81803258 and 82003475) and Jiangsu Province's Science and Technology Project (Grant No. BE2020722).

## Conflict of interest

The authors declare that the research was conducted in the absence of any commercial or financial relationships that could be construed as a potential conflict of interest.

## Publisher's note

All claims expressed in this article are solely those of the authors and do not necessarily represent those of their affiliated organizations, or those of the publisher, the editors and the reviewers. Any product that may be evaluated in this article, or claim that may be made by its manufacturer, is not guaranteed or endorsed by the publisher.
